# Supplementary material for: Comprehensive benchmarking of metagenomic binning tools reveals key factors for improved genome recovery
Source: Nat Commun. 2026 Apr 14;17:3467. doi: 10.1038/s41467-026-71521-w (PMC13079874; doi:10.1038/s41467-026-71521-w)
Supplement: Supplementary file 1 — Supplementary Information [file 41467_2026_71521_MOESM1_ESM.pdf]

Supplementary Information

Comprehensive benchmarking of metagenomic binning tools reveals key factors for improved genome recovery

Jungyeon Kim[1], Nayeon Kim[1], Jun Hyung Cha[1], Junyeong Ma[1], and Insuk Lee[1,2]*

[1]Department of Biotechnology, College of Life Science and Biotechnology, Yonsei University, Seoul 03722, Republic of Korea

[2]DECODE BIOME Co., Ltd. Incheon 21983, Republic of Korea

*Corresponding author: Insuk Lee

Tel: +82-10-4186-8706, E-mail: insuklee@yonsei.ac.kr

Contents

1. Supplementary Figures 1-4

2. Supplementary Tables 1-5

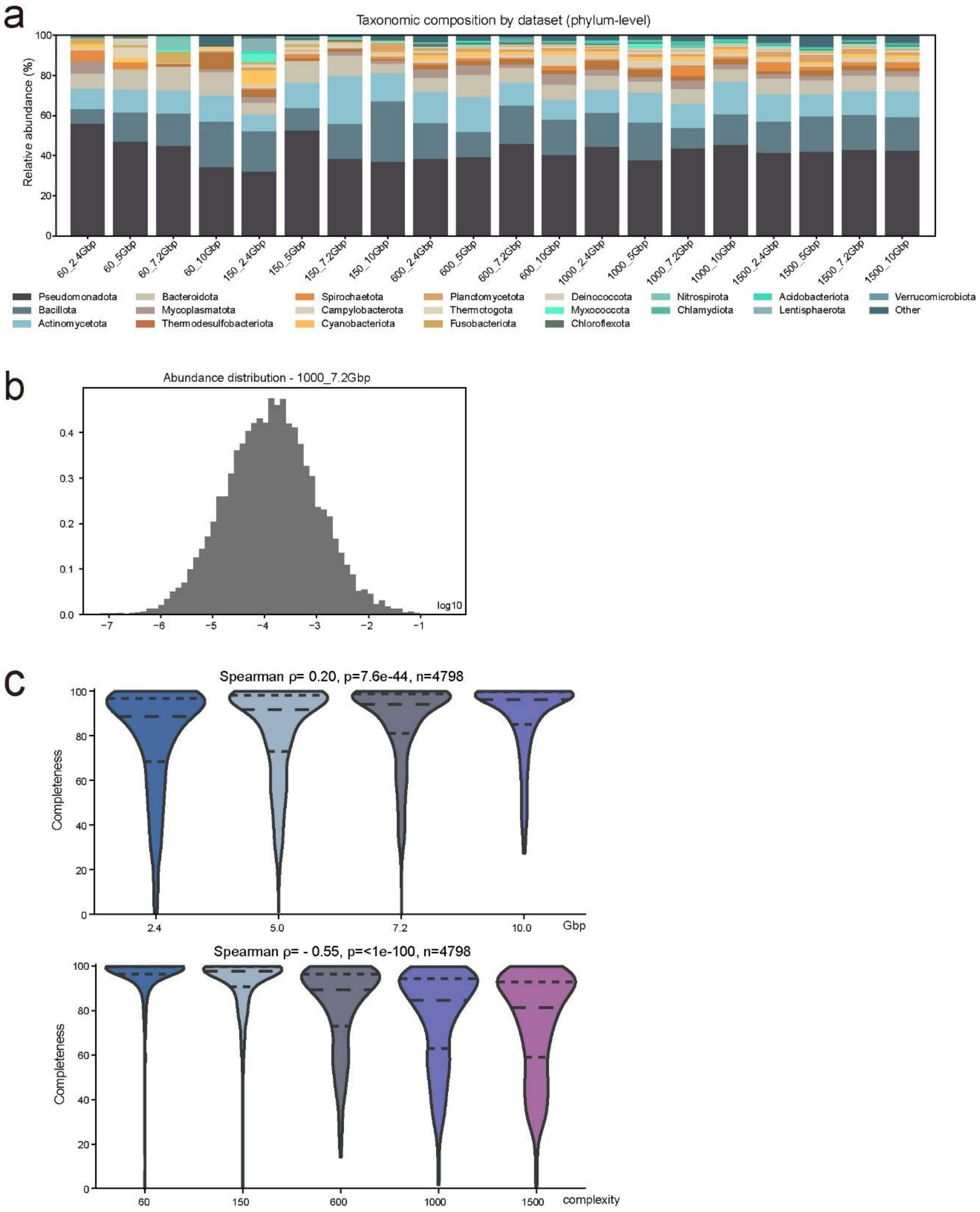

Supplementary Figure 1. Characteristics of the simulated datasets and benchmarking correlations. a, Phylum-level taxonomic composition of all simulated datasets generated with CAMISIM using a de novo community design. Each dataset shows broad taxonomic diversity, covering major bacterial phyla such as *Pseudomonadota, Bacillota,* and *Actinomycetota,* and reflects a compositionally diverse synthetic community. **b**, Representative abundance distribution for the 7.2 Gb simulated community, illustrating an approximately log-normal pattern spanning over six orders of magnitude. **c**, Correlations between sequencing depth or community complexity and the completeness of reconstructed MAGs across all simulated datasets. Completeness scores increased with sequencing depth but decreased with community complexity (Spearman's ρ and *p*-values shown in the panels).

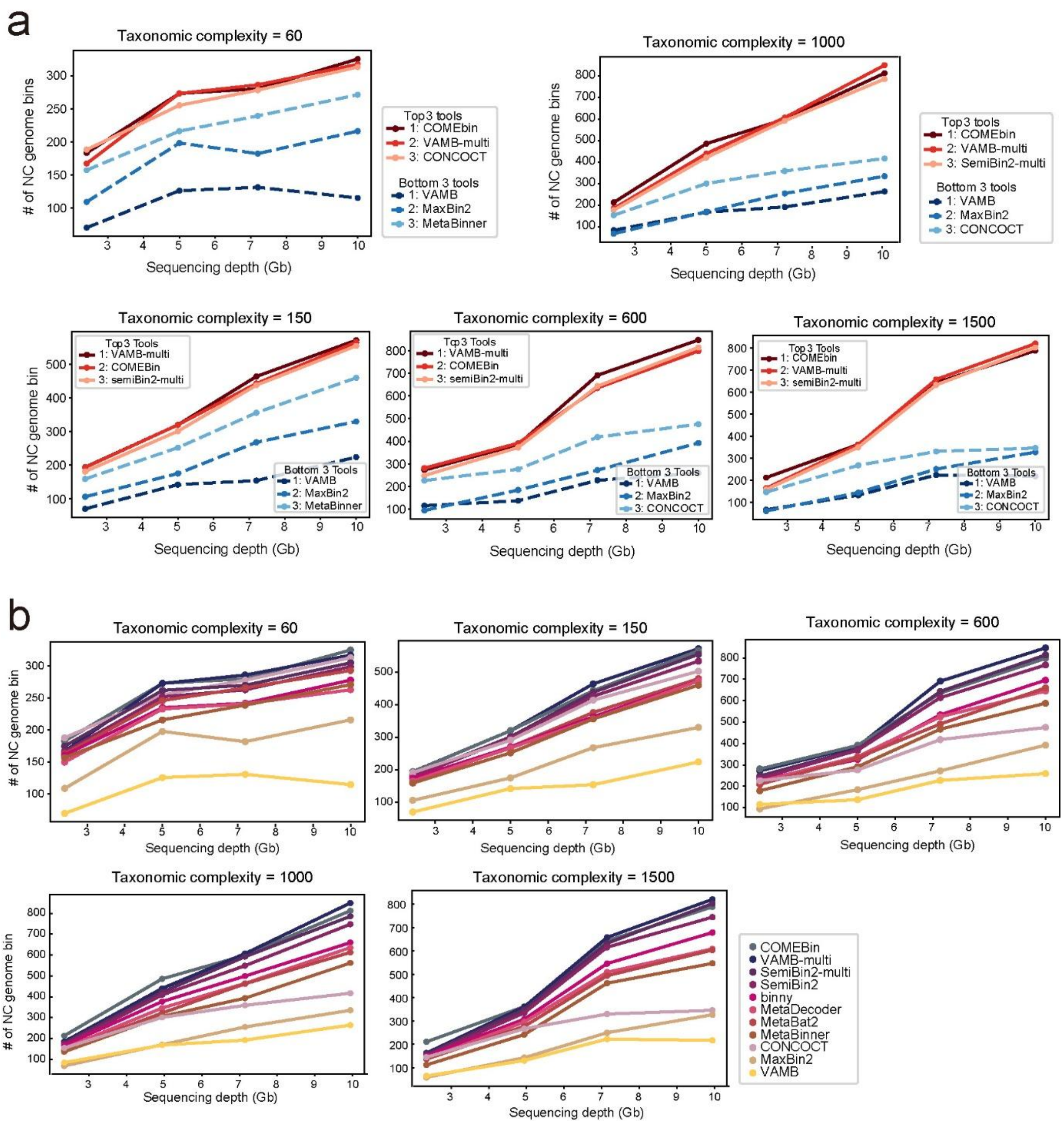

Supplementary Figure 2. Impact of sequencing depth on binning performance in simulation datasets. a, Number of Near Complete MAGs reconstructed across increasing sequencing depths in the in-house simulation datasets. Only the top three and bottom three performing binning tools at each depth are shown. Each dataset contains 60, 150, 600, 1,000, or 1,500 genomes per sample. **b**, Same analysis as in panel a, but including all binning tools.

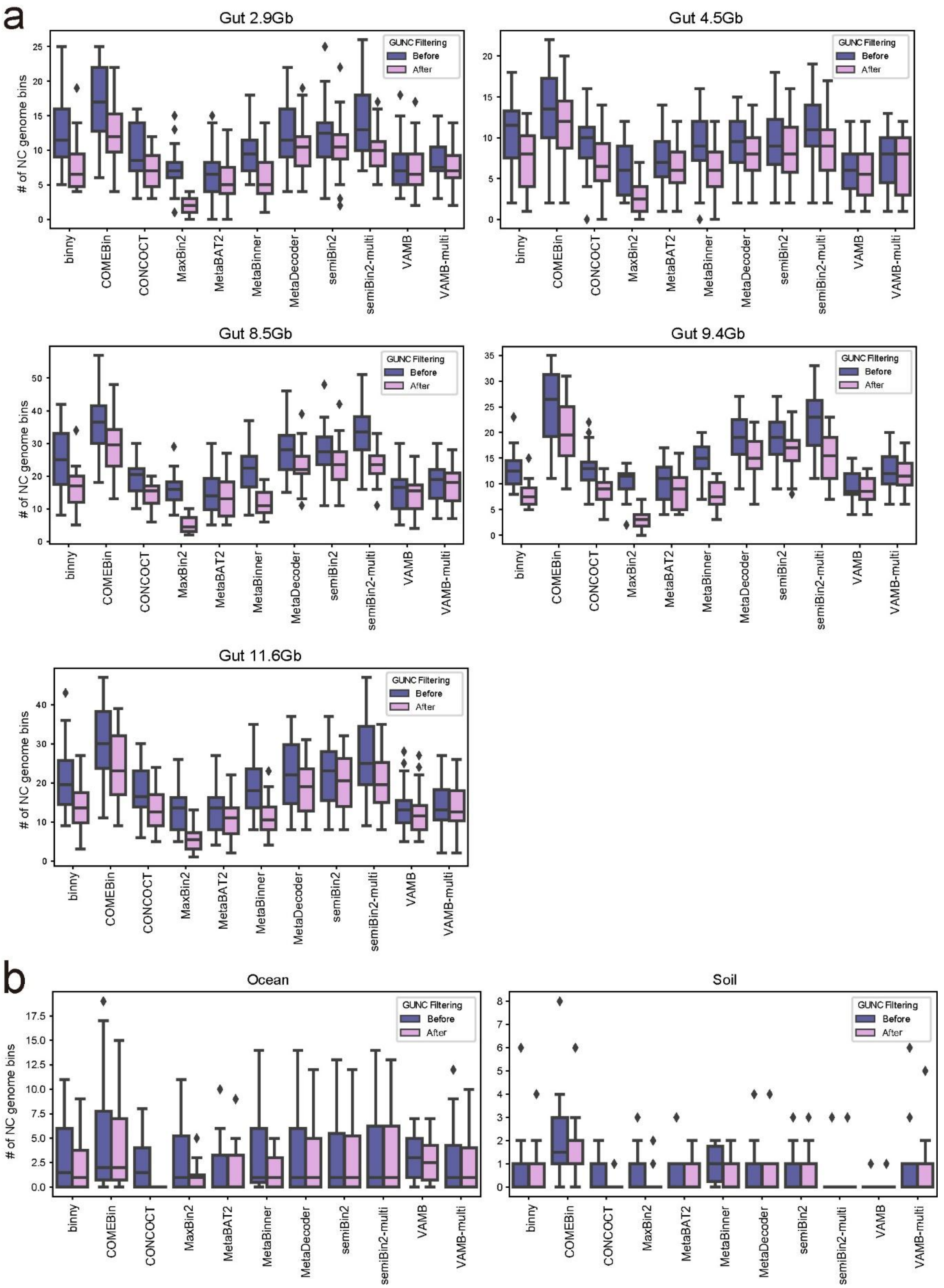

Supplementary Figure 3. Effect of GUNC filtering on NC MAG recovery across real microbiome datasets. Difference in NC MAG counts before and after GUNC filtering for each binning tool: **a**, across gut datasets (2.9Gb, 4.5Gb, 8.5Gb, 9.4Gb, 11.6Gb), **b**, ocean, and soil datasets.

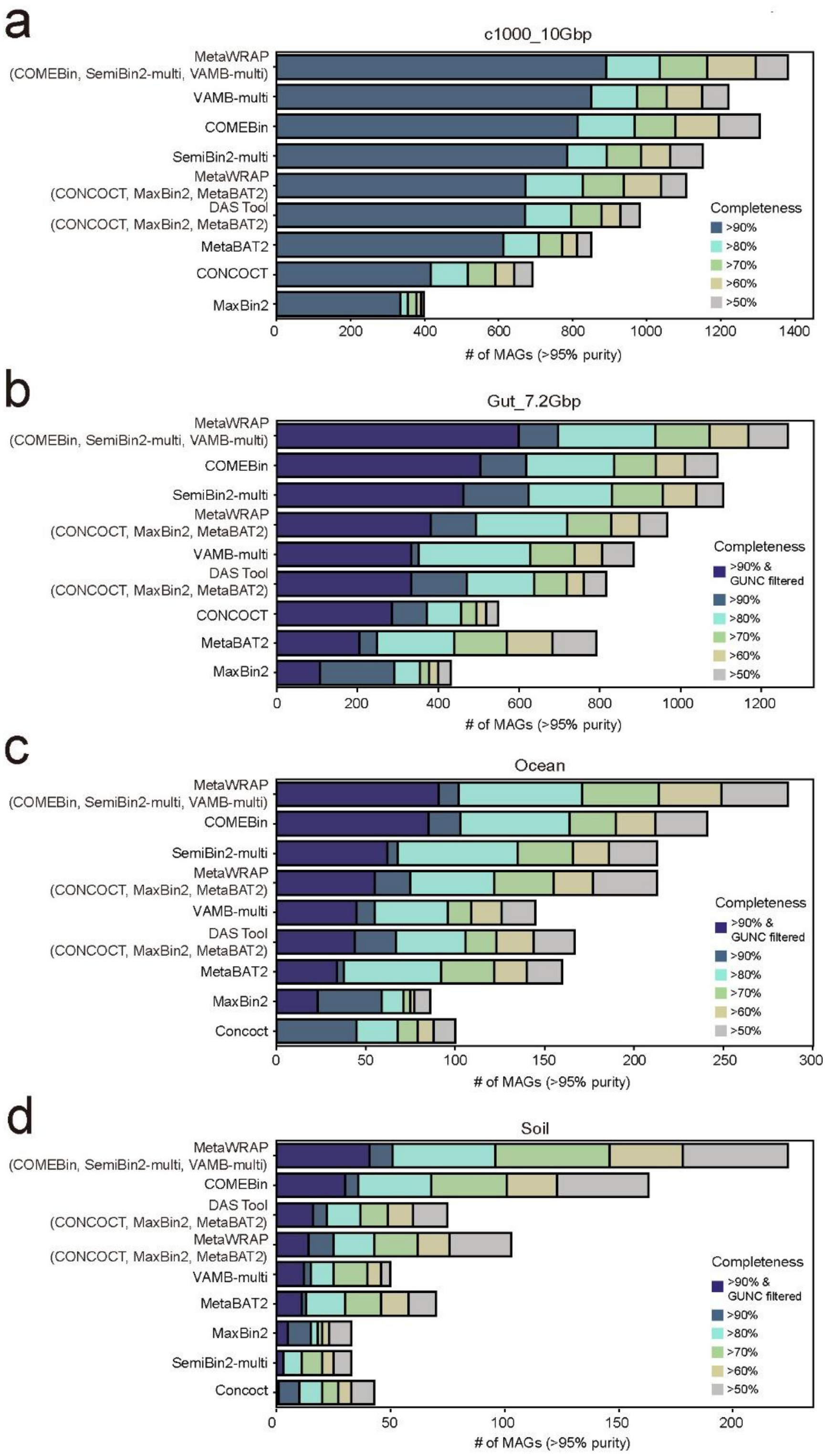

Supplementary Figure 4. Comparative evaluation of binning and refinement strategies across simulated and real metagenomic datasets. a–d, Comparison of MAG counts produced by six individual binning tools and their combinations through refinement across four datasets: **a**, c1000_10Gb; **b**, gut_7.2Gb; **c**, ocean; **d**, soil. All MAGs with contamination under 5% were included and grouped by completeness thresholds of 50%, 60%, 70%, 80%, and 90%. For real datasets, an additional category includes MAGs that meet the 90% completeness threshold and pass the GUNC filter (CSS score < 0.45).

Supplementary Table 1. Summary of binning tools

**Program**	**Version**	**DL**	**Input Information**	**Data processing & integration strategy**	**Type of integration**	**Clustering algorithm**	**COL**
binny	2.2.15	x	k-mer composition, coverage, SCG	Concatenation followed by non-linear projection (openTSNE)	Projection-based	HDBSCAN	semi
COMEBin	1.0.4	o	k-mer composition, coverage, SCG	Multi-view contrastive learning via k-mer encoders	Neural Network	Density-based clustering (Leiden)	un
CONCOCT	1.1.0	x	k-mer composition, coverage	Concatenation followed by linear projection (PCA)	Projection-based	Probabilistic model(GMM)	un
MaxBin2	2.2.7	x	k-mer composition, coverage, SCG	Probabilistic integration through feature-wise modeling and multiplication	Probabilistic Modeling	Probabilistic model (EM)	un
MetaBAT2	2.15.0	x	k-mer composition, coverage	Geometric mean of normalized similarity scores	Probabilistic Modeling	Graph-based Modified Label Propagation	un
MetaBinner	1.4.4	x	k-mer composition, coverage, SCG	Score matrix ensemble via majority voting	Ensemble-based	Partial seed k-means clustering	semi
MetaDecoder	1.0.16	x	k-mer composition, coverage, SCG	Concatenation followed by semi-supervised probabilistic modeling	Probabilistic Modeling	Probabilistic model (DPGMM + GMM)	semi
SemiBin2	1.4.0	o	k-mer composition, coverage, SCG, must-link, cannot-link	Concatenation followed by Siamese neural network embedding	Neural Network	Density-based clustering (DBSCAN)	self
VAMB	3.0.9	o	k-mer composition, coverage	Concatenation followed by variational autoencoding (VAE)	Neural Network	Iterative k-medoid clustering	un

TNF, tetranucleotide frequency; SCG, single-copy marker gene; DL, Deep learning

COL, category of learning; Semi, semi-supervised; un, unsupervised; self, self-supervised

Supplementary Table 2. Summary of real metagenome sequencing datasets for benchmarking

Dataset	Project No.	Avg. seq. depth (Gbp, after pre-processing)	Read format
single_7Gb	PRJNA530339	7.0	single
single_10Gb	PRJNA530339	10.0	single
gut_2.9Gb	PRJNA797994	2.9	paired
gut_4.5Gb	PRJEB27005	4.5	paired
gut_7.2Gb	PRJEB33013	7.2	paired
gut_8.5Gb	PRJEB39223	8.5	paired
gut_9.4Gb	PRJDB4525	9.4	paired
gut_11.6Gb	PRJNA624763	11.6	paired
ocean	PRJNA273799	2.2	paired
soil	PRJNA261849	4.0	paired

No., Number; Avg., Average

Supplementary Table 3. Measured community complexity of CAMI2 human datasets

Dataset	Sample	Complexity	Sequencing depth (bp)
CAMI_gitrack	0	69	4,624,411,070
CAMI_gitrack	1	63	4,625,028,328
CAMI_gitrack	2	46	4,625,007,230
CAMI_gitrack	3	57	4,624,967,696
CAMI_gitrack	4	52	4,624,817,273
CAMI_gitrack	5	24	4,624,502,654
CAMI_gitrack	9	56	4,625,887,009
CAMI_gitrack	10	59	4,624,565,746
CAMI_gitrack	11	82	4,625,197,929
CAMI_gitrack	12	35	4,625,028,328
CAMI_oral	6	89	4,997,272,800
CAMI_oral	7	179	4,997,304,300
CAMI_oral	8	163	4,998,814,200
CAMI_oral	13	164	4,999,308,300
CAMI_oral	14	141	4,999,810,800
CAMI_oral	15	165	4,999,836,000
CAMI_oral	16	320	4,998,828,000
CAMI_oral	17	143	4,999,739,700
CAMI_oral	18	150	4,999,780,200
CAMI_oral	19	274	4,996,806,000
CAMI_airway	4	255	4,999,463,400
CAMI_airway	7	186	4,999,767,600
CAMI_airway	8	135	4,999,502,400
CAMI_airway	9	192	4,999,780,500
CAMI_airway	10	201	4,999,777,500
CAMI_airway	11	76	4,999,733,400
CAMI_airway	12	96	4,999,759,200
CAMI_airway	23	98	4,999,782,300
CAMI_airway	26	212	4,999,094,100
CAMI_airway	27	195	4,999,782,300

Supplementary Table 4. Computational cost (gut_7.2Gb, 5 core CPU)

**Binning tool**	**Run time (min)**	**Memory (GB)**
binny	158.66	7.49
COMEBin	247.22	5.73
COMEBin(GPU)	61.25	5.41
CONCOCT	26.94	0.93
MaxBin2	16.20	0.89
MetaBAT2	5.02	2.13
MetaBinner	49.80	0.89
MetaDecoder	4.81	0.89
SemiBin2	35.77	3.35
SemiBin2(multi)	39.48	4.70
VAMB	4.86	0.36
VAMB(multi)	5.19	6.63

Supplementary Table 5. Computational cost (gut_7.2Gb, 5 core CPU) for refining bins from COMBEBin, SemiBin2-multi, and VAMB-multi

Refinement tool	Run time (min)	Memory (GB)
DASTool	7.91	0.49
MAGScoT	2.47	0.29
MetaWRAP	84.80	11.98
