# Supplementary material for: Comprehensive benchmarking of metagenomic binning tools reveals key factors for improved genome recovery
Source: Nat Commun. 2026 Apr 14;17:3467. doi: 10.1038/s41467-026-71521-w (PMC13079874; doi:10.1038/s41467-026-71521-w)
Supplement: Supplementary file 3 — Reporting Summary [file 41467_2026_71521_MOESM3_ESM.pdf]

nature portfolio

Corresponding author(s): Insuk Lee

Last updated by author(s): Feb 11, 2026

Reporting Summary

Nature Portfolio wishes to improve the reproducibility of the work that we publish. This form provides structure for consistency and transparency in reporting. For further information on Nature Portfolio policies, see our Editorial Policies and the Editorial Policy Checklist.

Statistics

For all statistical analyses, confirm that the following items are present in the figure legend, table legend, main text, or Methods section.

n/a	Confirmed	
☐	☒	The exact sample size (*n*) for each experimental group/condition, given as a discrete number and unit of measurement
☐	☒	A statement on whether measurements were taken from distinct samples or whether the same sample was measured repeatedly
☐	☒	The statistical test(s) used AND whether they are one- or two-sided<br>*Only common tests should be described solely by name; describe more complex techniques in the Methods section.*
☒	☐	A description of all covariates tested
☒	☐	A description of any assumptions or corrections, such as tests of normality and adjustment for multiple comparisons
☐	☒	A full description of the statistical parameters including central tendency (e.g. means) or other basic estimates (e.g. regression coefficient) AND variation (e.g. standard deviation) or associated estimates of uncertainty (e.g. confidence intervals)
☐	☒	For null hypothesis testing, the test statistic (e.g. *F*, *t*, *r*) with confidence intervals, effect sizes, degrees of freedom and *P* value noted<br>*Give P values as exact values whenever suitable.*
☒	☐	For Bayesian analysis, information on the choice of priors and Markov chain Monte Carlo settings
☒	☐	For hierarchical and complex designs, identification of the appropriate level for tests and full reporting of outcomes
☒	☐	Estimates of effect sizes (e.g. Cohen's *d*, Pearson's *r*), indicating how they were calculated

Our web collection on statistics for biologists contains articles on many of the points above.

Software and code

Policy information about availability of computer code

Data collection	Synthetic datasets were generated using CAMISIM (v1.3.0) with default parameters and de novo community design using bacterial isolate genomes from NCBI RefSeq (https://www.ncbi.nlm.nih.gov/refseq/).<br>All metagenomic datasets used in this study were obtained from public repositories. Human gut, marine, and soil datasets were downloaded from the NCBI Sequence Read Archive (SRA).<br>No software was used for the data collection process.
Data analysis	Assemblers used include MEGAHIT v1.2.9 and metaSPAdes v3.13.0<br>Reads were aligned using Bowtie2 v2.5.3<br>Genome binning was performed using SemiBin2 v1.4.0, VAMB v3.0.9, MetaBAT2 v2.15, MaxBin2 v2.2.7, CONCOCT v1.1.0, MetaDecoder v1.0.16, MetaBinner v1.4.4, binny v2.2.15, and COMEBin v1.0.4<br>Bin refinement was conducted using MetaWRAP v1.3.2, DAS Tool v1.1.6, and MAGScoT v1.0.0<br>Genome bin quality was assessed using AMBER v2.0.0, CheckM2 v0.1.2, and GUNC v1.0.6<br>Species-level clustering and phylogenetic analysis were performed using dRep v3.4.5, Mash v2.2, fastANI v1.33, GTDB-Tk v2.1.1, IQ-TREE v2.3.6, and visualized with iTOL (https://itol.embl.de/)<br>Data analysis and figure generation were conducted using custom Python and R scripts, including ggplot2, seaborn, matplotlib, and UpsetPlot.<br>All commands and custom code used for benchmarking metagenomic binning tools are publicly available at https://github.com/netbiolab/Benchmarking_metagenomic_binners.

For manuscripts utilizing custom algorithms or software that are central to the research but not yet described in published literature, software must be made available to editors and reviewers. We strongly encourage code deposition in a community repository (e.g. GitHub). See the Nature Portfolio guidelines for submitting code & software for further information.

Data

Policy information about availability of data

All manuscripts must include a data availability statement. This statement should provide the following information, where applicable:

- Accession codes, unique identifiers, or web links for publicly available datasets
- A description of any restrictions on data availability
- For clinical datasets or third party data, please ensure that the statement adheres to our policy

All benchmarking data is publicly available. The simulated benchmarking data generated in this study have been deposited in the Zenodo database under accession code 10.5281/zenodo.18916168. The processed benchmarking results data are available at https://github.com/netbiolab/Benchmarking_metagenomic_binners. The CAMI II Toy Human Microbiome Project data used in this study are available in PUBLISSO with DOIs 10.4126/FRL01-006425518, and on the CAMI data portal (https://data.cami-challenge.org/participate). The real metagenome datasets used in this study are available in the European Nucleotide Archive (ENA) under accession code PRJNA530339, PRJNA797994, PRJEB27005, PRJEB33013, PRJEB39223, PRJDB4525, PRJNA624763, PRJNA273799, PRJNA261849. All data supporting the findings described in this manuscript are available in the article and in the Supplementary Information. Source data are provided with this paper.

Research involving human participants, their data, or biological material

Policy information about studies with human participants or human data. See also policy information about sex, gender (identity/presentation), and sexual orientation and race, ethnicity and racism.

Reporting on sex and gender	Not applicable.
Reporting on race, ethnicity, or other socially relevant groupings	Not applicable.
Population characteristics	Not applicable.
Recruitment	Not applicable.
Ethics oversight	Not applicable.

Note that full information on the approval of the study protocol must also be provided in the manuscript.

Field-specific reporting

Please select the one below that is the best fit for your research. If you are not sure, read the appropriate sections before making your selection.

☒ Life sciences ☐ Behavioural & social sciences ☐ Ecological, evolutionary & environmental sciences

For a reference copy of the document with all sections, see nature.com/documents/nr-reporting-summary-flat.pdf

Life sciences study design

All studies must disclose on these points even when the disclosure is negative.

Sample size	The number of samples per dataset was determined based on the availability of public metagenomic datasets and the design of the simulated data. The final sample sizes were also chosen to balance computational efficiency and practical feasibility. No formal power calculation was performed.
Data exclusions	There was no data exclusion.
Replication	All benchmarking analyses were replicated across multiple simulated and real-world datasets to ensure consistency.
Randomization	There was no randomization.
Blinding	There was no blinding design in this study.

Reporting for specific materials, systems and methods

We require information from authors about some types of materials, experimental systems and methods used in many studies. Here, indicate whether each material, system or method listed is relevant to your study. If you are not sure if a list item applies to your research, read the appropriate section before selecting a response.

Materials & experimental systems

n/a	Involved in the study
☒	☐ Antibodies
☒	☐ Eukaryotic cell lines
☒	☐ Palaeontology and archaeology
☒	☐ Animals and other organisms
☒	☐ Clinical data
☒	☐ Dual use research of concern
☒	☐ Plants

Methods

n/a	Involved in the study
☒	☐ ChIP-seq
☒	☐ Flow cytometry
☒	☐ MRI-based neuroimaging

Plants

Seed stocks

Report on the source of all seed stocks or other plant material used. If applicable, state the seed stock centre and catalogue number. If plant specimens were collected from the field, describe the collection location, date and sampling procedures.

Novel plant genotypes

Describe the methods by which all novel plant genotypes were produced. This includes those generated by transgenic approaches, gene editing, chemical/radiation-based mutagenesis and hybridization. For transgenic lines, describe the transformation method, the number of independent lines analyzed and the generation upon which experiments were performed. For gene-edited lines, describe the editor used, the endogenous sequence targeted for editing, the targeting guide RNA sequence (if applicable) and how the editor was applied.

Authentication

Describe any authentication procedures for each seed stock used or novel genotype generated. Describe any experiments used to assess the effect of a mutation and, where applicable, how potential secondary effects (e.g. second site T-DNA insertions, mosiacism, off-target gene editing) were examined.
